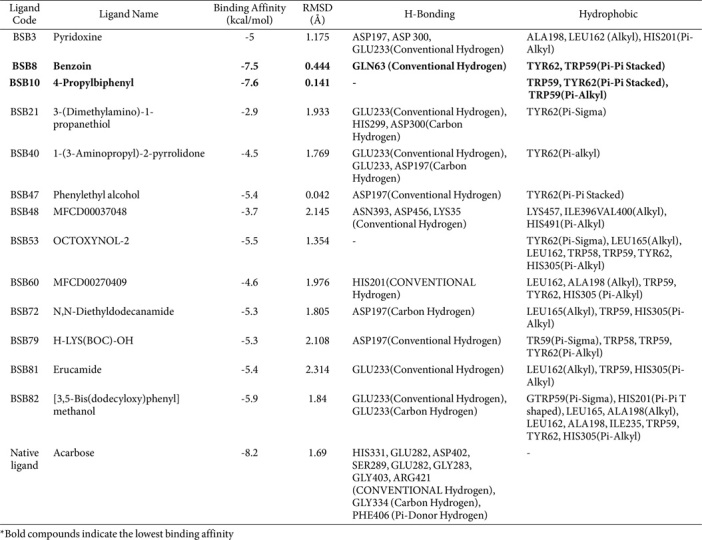# Efficacy of Endophytic Bacterium *Serratia marcescens* B.SB 1.1 associated with Sea Fern (*Acrostichum aureum* L.) as an Antidiabetic Agent

**DOI:** 10.4014/jmb.2412.12031

**Published:** 2025-04-27

**Authors:** Tetty Marta Linda, Dinda Putri Maisyaroh, Azizul Berlyansah, Balqis Juanne Tasliyah, Erwina Juliantari, Delita Zul, Bernadeta Leni Fibriarti, Asih Rahayu Ajeng Agesti, Yuli Haryani

**Affiliations:** 1 Department of Biology, Faculty of Mathematics and Natural Sciences, University of Riau, Kampus Bina Widya Km. 12, 5, Simpang Baru Pekanbaru 28293, Indonesia; 2 Department of Biology Education, Faculty of Teacher Training and Education, University of Riau, Kampus Bina Widya Km. 12, 5, Simpang Baru Pekanbaru 28293, Indonesia; 3 Department of Chemistry, Faculty of Mathematics and Natural Sciences, University of Riau, Kampus Bina Widya Km. 12, 5, Simpang Baru, Pekanbaru 28293, Indonesia

**Keywords:** *Acrostichum aureum* L., α-amylase inhibitors, endophytic bacteria, phytochemicals

## Abstract

Diabetes mellitus (DM) is a primary global health concern, often progressing unnoticed until complications arise. Current antidiabetic therapies primarily aim to inhibit the α-amylase enzyme, thereby reducing blood glucose levels. Some medicinal plants are proven to be symbiotic with endophytic bacteria that produce bioactive compounds capable of inhibiting α-amylase activity. This study investigated the potential of endophytic bacteria isolated from the stem of the sea fern (*Acrostichum aureum* L.) to act as α-amylase inhibitors, using both *in vitro* and in silico studies. Phytochemical analysis of both the stem extract and cultured bacterial isolates showed the presence of alkaloids, flavonoids, and saponins. Isolate B.SB 1.1 was identified as *Serratia marcescens* based on 16S rRNA sequencing. The α-amylase inhibition assay demonstrated the strain as showing significant inhibitory activity, with 32.57% inhibition at 2% starch substrate concentration. In silico docking studies using LC-MS data predicted 4-propylbiphenyl and benzoin as compounds with the lowest binding energy to α-amylase, suggesting their potential as effective inhibitors. These findings highlight the efficacy and therapeutic potential of endophytic strain *S. marcescens* B.SB 1.1 as a novel antidiabetic agent.

## Introduction

The global prevalence of diabetes mellitus (DM) has been a point of concern with a reported incidence rate of 7.5% (374 million cases) in 2019, a level projected to reach 8.0% (454 million cases) by 2030 [1]. Diabetes is a metabolic disorder characterized by persistent hyperglycemia along with disturbances in carbohydrate, lipid, and protein metabolism, stemming from either a relative or absolute deficit in insulin secretion or action [2]. Diabetes mellitus is not curable, but it can be controlled by keeping sugar levels within a normal range (80-120 mg/dl) [3].

A pivotal aspect of diabetes treatment involves the inhibition of α-amylase found in the saliva and pancreatic juice [4]. This enzyme plays a crucial role in the hydrolysis of carbohydrates (oligosaccharides and disaccharides) into monosaccharides, and its action is targeted by antidiabetic therapeutics to reduce glucose absorption from the intestinal lumen into the bloodstream [5]. Notably, active compounds derived from medicinal plants have potential as α-amylase inhibitors, offering a promising avenue for the development of natural antidiabetic agents.

The sea fern plant (*Acrostichum aureum* L.) is commonly found in wetland areas, such as mangrove forests and coastal areas in tropical and subtropical regions [6]. Traditionally, sea ferns are used to treat rheumatism, sore throat, gastric ache, chest pain, ulcers, and wounds [7]. Sea ferns contain phytochemical compounds, including flavonoids, saponins, terpenoids, and steroids [8]. These compounds play a role in various biological activities and have antimicrobial, antioxidant, antidiabetic, anticancer, and anti-inflammatory effects [9-13]. However, using plants as herbal medicine comes with various drawbacks, such as limited quantity, relatively long life cycle, and the exploitation of natural resources. Therefore, alternative sources of antidiabetic compounds are needed.

Endophytic bacteria have a symbiotic relationship with plant species, enabling them to synthesize bioactive compounds similar to the secondary metabolites of the host plant, and that can be utilized as raw materials in medicine [14, 15]. Moreover, medicinal plants are a potential source of microbes producing α-glucosidase enzyme inhibitors. By obtaining potential isolates from these plants, the production of α-amylase-inhibiting compounds for use as diabetes drugs can be done microbiologically, in higher amounts and with better quality, which can help with conservation efforts [16].

Exploring new sources of endophytic bacteria is crucial for discovering bioactive compounds with potential applications in the health sector. Endophytic bacterial isolates from sea fern leaf are known to contain alkaloids and saponins [17, 18]. The content of phytochemical compounds in isolates of endophytic bacteria *Lysinibacillus* spp. HSRN and *Enterobacter* spp. SSRP1 isolated from the stem of the *Combretum molle* plant showed positive results for flavonoids and tannins, as well as antibacterial activity against *Bacillus cereus*, *Staphylococcus aureus*, *Escherichia coli*, and *Pseudomonas aeruginosa* [19]. Isolates from rambutan plants (*Nephelium lappaceum* L.) produce α-amylase inhibitors, which have potential as anti-diabetic compounds [20]. Furthermore, C5 endophytic bacteria isolated from cinnamon bark (*Cinnamomum burmanii*) can produce flavonoids, tannins, and saponins, which have potential as anti-diabetic agents [21].

Currently, the development of antidiabetic drugs is still carried out using traditional methods and development procedures that can be time-consuming and costly, with a low success rate [22]. In silico methods offer a solution by enabling the efficient, focused development of targeted drugs based on highly promising compounds [23]. Moreover, in silico methods accelerate drug development through the screening of identified compounds for selection of the best drug candidates with optimal properties, thereby achieving clinical efficacy and minimizing clinical toxicity [24]. Some of the compounds identified as antidiabetics through in silico methods are colletotrichalactone A from endophytic fungi of *Morus* sp. [25]; erythrin from lichens [26]; and gypsogenin, adunctin C, sitosterol and stigmasterol from Philippine plants with activity against four protein targets of diabetes [27]. Following the selection of promising compounds for further study, a process facilitated by in silico methods, newly discovered natural medicines can then undergo *in vitro* or *in vivo* validation.

At present there is limited information about endophytic bacteria from sea fern plants. In this study we aimed to isolate and test the phytochemical content and α-amylase-inhibiting activity of crude extracts of endophytic bacteria from sea fern stems (*Acrostichum aureum* L.) We used *in vitro* and in silico methods to identify the ability of endophytic bacteria of sea fern stems as α-amylase inhibitors. In silico methods were used to examine their structures and binding energies to determine the interactions between endophytic bacteria-derived compounds and α-amylase. Our findings can potentially lead to the development of new antidiabetic drugs from the endophytic bacteria of sea fern stems to target the α-amylase enzyme.

## Materials and Methods

### Sample Collection and Endophytic Bacteria Isolation

Sea fern samples (*Acrostichum aureum* L.) were taken from the east coast of Sumatra Island, Indonesia, in Selatbaru Village, Bengkalis Regency, Riau Province. The selected plant samples were stems of mature, healthy sea ferns.

The samples were washed in sodium hypochlorite (NaOCl) solution for 6 min, rinsed with sterile water three times, and dried using a tissue. Samples were then cut to a size of 2 × 2 cm and placed into a Petri dish containing nutrient agar (NA) medium and incubated for 48 h at room temperature. The final rinse water was used as a control by pouring 1 ml into the NA medium using the pour plate technique followed by incubation for 24 h. Isolation of endophytic bacteria was successful if the control medium contained no growing microorganisms.

### Purification and Characterization of Endophytic Bacteria

Endophytic bacterial isolates growing around the stem samples were purified in an NA medium using the streak quadrant technique to obtain a single colony. Each growing bacterial colony with a different morphology was separated. The purified bacterial isolates were then stored in a slanted NA medium in a refrigerator at 4°C for use in the next stage. Macroscopic characterization of the morphology of endophytic bacteria included observations of color, margins, elevation, and colony size. The microscopic description was carried out by Gram test on each endophytic bacterial isolate.

### Production of Secondary Metabolites of Endophytic Bacteria

As much as 10% (v/v) of each inoculum of the endophytic bacteria isolates with a total population of 10^8^ CFU/ml was collected and put in 90 ml of nutrient broth (NB). Then, the media was incubated in an incubator shaker at 150 rpm and 30°C for 72 h [17]. The endophytic bacterial culture was centrifuged at 3,000 ×*g* for 20 min to separate the supernatant and pellet parts.

### Phytochemical Test

Phytochemical tests were carried out on sea fern stem extracts and endophytic bacteria crude extracts. Sea fern stems were washed, weighed as much as 1 g, and mixed with 5 ml of distilled water. The solution was heated for 2 min until it boiled, and then filtered to obtain the extract. The supernatant of the endophytic bacteria of sea fern stems was used for phytochemical tests.

**Alkaloid.** Each sea fern stem extract and 5 ml of endophytic bacteria supernatant were added with 2 ml chloroform and 2 ml of ammonia, and then filtered. The filtrate obtained was then added with five drops of concentrated H_2_SO_4_ and shaken until two layers were formed. The top layer was transferred to a new test tube, and five drops of Mayer's reagent were added. The formation of a white precipitate indicated a positive result in the alkaloid test after adding Mayer's reagent [17].

**Flavonoid.** Each sea fern stem extract and 5 ml of endophytic bacteria supernatant was added with 5 g of magnesium (Mg) powder and five drops of concentrated HCl until the color changed. The flavonoid test indicates a positive result by a change in the color of the solution to yellow [28].

**Saponin.** Each sea fern stem extract and 5 ml of endophytic bacteria supernatant were added to 10 ml of sterile distilled water and shaken for 1 min. The formation of stable foam for 10 min indicates a positive result in the saponin test [17].

**Tannin.** Each sea fern stem extract and 5 ml of endophytic bacteria supernatant were added to ten drops of 10%iron (III) chloride (FeCl_3_) solution. Positive results in the tannin test are indicated by a change in the color of the solution to black-green [29].

**Steroid-Terpenoid.** Each sea fern stem extract and 5 ml of endophytic bacteria supernatant were added with ten drops of glacial CH_3_COOH plus two drops of concentrated H_2_SO_4_ solution, then shaken gently and incubated for 1-2 min. A positive result in the steroid test is indicated by a change in the color of the solution to green-blue, whereas if a red color change is formed in the solution, it shows a positive result in the terpenoid test [17].

### Molecular Identification of Endophytic Bacteria

An endophytic bacterial inoculum with a population of 10^8^ CFU/ml was put into a 1.5 ml Eppendorf tube and extracted according to the instructions of the PrestoTM Mini gDNA Bacteria Kit (Geneaid, Taiwan). The results of total DNA isolation were examined using electrophoresis on agarose gel (1%) in 1x Tris-Borate-EDTA (TBE) solution for 45 min at 50 volts. After that, it was visualized using a UV transilluminator and photographed using an Olympus digital camera (Japan) with a UV filter.

### 16S rRNA Gene Amplification by PCR Technique

Endophytic bacteria were identified molecularly using 16S rRNA. The tests were carried out following the instructions of the PCR Presto^TM^ Mini gDNA Bacteria Kit (Geneaid), with forward and reverse primers using universal primers, namely 8F (5'-AGA GTT TGA TCC TGG CTC AG-3') and 1492R (5'-GGT TAC CTT GTT ACG ACT T-3'). The PCR process was carried out for 35 cycles, starting with pre-PCR at 94°C for 2 min, with each cycle consisting of denaturing DNA templates at 94°C for 30 s followed by annealing/primer attachment at 49.85°C for 1.30 min, and an extension for 1 min at 72°C. Finally, the post-PCR step was carried out at 72°C for 5 min. The integrity of the PCR product was checked using electrophoresis. The PCR results of the samples were analyzed based on the fragment sequence of the 16S rRNA gene. Nucleotides were sorted by sending 50 μl of PCR product to PT Genetics Science Indonesia in Jakarta.

### α-Amylase Inhibitor Test from Secondary Metabolites of Endophytic Bacteria

Testing for α-amylase inhibitors was conducted with a modified method [30]. To test a sample as an inhibitor (S), 0.5 units/ml of the α-amylase enzyme dissolved in phosphate buffer at pH 6.9 was added to 0.5 ml of the sample and then incubated for 10 min at 25°C. Then, 1 ml of 1% and 2% starch solution was added, followed by incubation for 10 min at 25°C. The same treatment was carried out for the negative control (C) without adding samples. After incubation for 10 min, the reaction was stopped by adding 2 ml of dinitro salicylic acid (DNS) reagent to the control (C) and sample (S) and heated using a water bath for 5 min. Absorbance was measured using a spectrophotometer with a wavelength of 540 nm. The material used as a positive control was acarbose. The following formula was used to calculate the percentage of inhibition:

Inhibition (%) = ((C-S))/C × 100%.

Note:

K = absorbance of enzyme control + substrate + liquid medium

S = absorbance of enzyme control + substrate + inhibitor

### Protein and Ligand Preparation for In Silico Analysis

Compounds obtained from endophytic extracts by LC-MS were selected as a ligand and protein target and analyzed using the SwissTargetPrediction web server (http://www.swisstargetprediction.ch/). Chemical structures of the ligand were obtained in SMILES format from PubChem (https://pubchem.ncbi.nlm.nih.gov/). The protein structure, a prerequisite in docking studies, was downloaded from the Protein Data Bank web server (https://www.rcsb.org/). The protein target in this study was α-amylase (PDB ID 1B2Y), which represented the 3D structure of human pancreatic α-amylase in complex with Acarbose at 3.20 Å resolution. The structure of protein from the protein data bank was prepared using the PyMOL application to remove hydrogen, heteroatoms, and water molecules [31].

### Bioactivity Prediction

Compounds of endophytic bacteria were analyzed for bioactivity prediction related to α-amylase-inhibitor activity using the PASS Online web server with parameters Pa > Pi and Pa > 0.3 [32]. Compounds meeting the parameters were then subjected to pharmacokinetics analysis.

### Pharmacokinetics and Drug-Likeness Analysis

The pkCSM and SwissADME webservers were used to perform pharmacokinetics analysis and drug-likeliness using Lipinski's Rule of Five [33]. The analysis was carried out by copying the ligands' Canonical SMILES on the pkCSM website (https://biosig.lab.uq.edu.au/pkcsm) to gather information on pharmacokinetic and toxicity features [34]. Toxicity was further assessed using the Pro-Tox II website (https://tox-new.charite.de) concerning carcinogenic, mutagenic effects, LD50, and toxicity classifications [35].

### Molecular Docking Studies

Ligands that fulfilled Lipinski's rule were used for docking study. Geometric optimization of the ligands was done and saved in pdb format. This molecular docking study was implemented using Pyrx application, and before the binding between ligand and protein, the ligand was validated using the Open Babel application. The binding of ligand and target protein was done by setting the grid box of the target protein. For illustration and consideration of molecular tethering score and best ligand position, various ligand-protein interactions were visualized using Discovery Studio Visualizer [36].

## Results

### Isolation and Characterization of Endophytic Bacteria

Isolation of endophytic bacteria using the direct technique was indicated by the growth of endophytic bacteria around the sample. The purification results of endophytic bacteria from samples of sea fern stems (*Acrostichum aureum* L.) obtained isolates of endophytic bacteria with an incubation time of 24 h on NA medium. The purified endophytic bacteria were then rejuvenated and characterized macroscopically and microscopically. The characterization results of selected endophytic bacteria from sea fern stems are shown in Table 1.

**Fig. 1:**
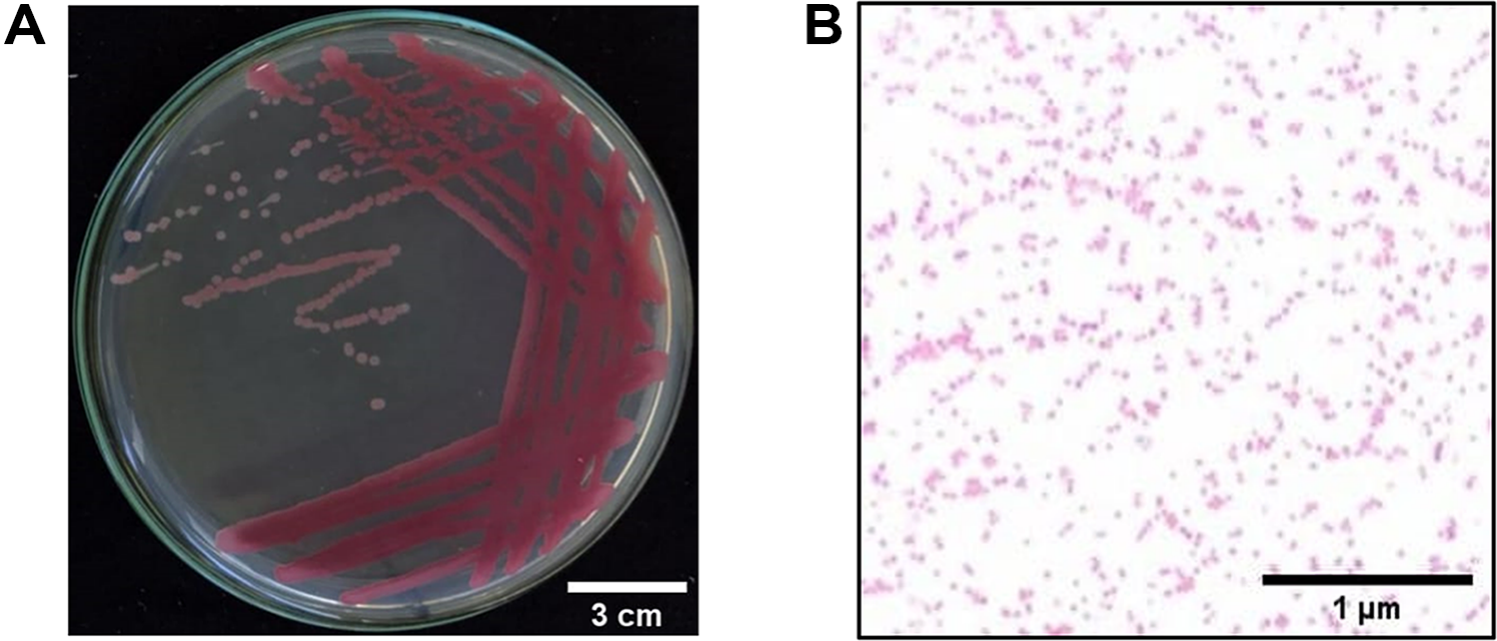
Isolate B.SB 1.1 of endophytic bacteria from sea fern stem (*A. aureum* L.). (**A**) Morphology of colony which shows the elevation and the edges of colony. (**B**) Gram staining of colony shows purplish-red color.

The morphology technique and Gram staining were used to identify the bacterial strains. The results indicated that isolate B.SB 1.1 was gram-negative, bacillus-shaped, and had a purplish-red color. Elevation of bacterial colonies on the agar surface implies the degree of colony expansion. The colonies of isolate B.SB 1.1 had a convex elevation and the margins of the colonies were entire (Fig. 1).

### Identification of Potential Endophytic Bacteria

A phylogenetic analysis leveraging 16S ribosomal RNA (rRNA) gene sequences is widely recognized as a pivotal method for elucidating bacterial phylogeny and taxonomic relationships. This study focuses on the molecular identification of endophytic bacterial isolates extracted from sea fern stems collected from the eastern coastline of Sumatra, specifically within Selatbaru Village, to categorize these bacteria based on their phylogenetic characteristics. The methodology employed involved the molecular characterization of the isolates through the amplification and subsequent sequencing of the 16S rRNA gene, with the sequences obtained being subjected to comparative analysis using the Basic Local Alignment Search Tool (BLAST) to ascertain their closest phylogenetic relatives.

The sequencing of the 16S rRNA gene from the B.SB 1.1 isolate yielded a sequence length of approximately 1,408 bp. Utilizing the 16S rRNA gene database, a similarity search indicated that the isolate exhibited a taxonomic alignment within the *Serratia* genus, showing the highest sequence homology to *S. marcescens*, as detailed in Table 2. This identification underscores the significance of 16S rRNA gene-based phylogenetic frameworks in accurately determining the lineage and relatedness of bacterial isolates derived from environmental samples.

**Fig. 2:**
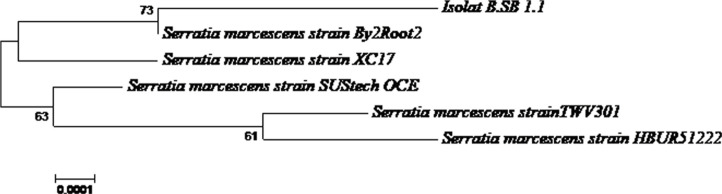
Phylogenetic tree based on the analysis of partial 16S rRNA gene sequences from isolate B.SB 1.1 that was constructed using reference sequences of Serratia from the NCBI database. Phylogenetic tree show isolate B.SB 1.1 closest to *Serratia marcescens*.

A phylogenetic tree was constructed using MEGA X software and the bootstrap algorithm. The results of phylogenetic tree reconstruction showed that isolate B.SB 1.1 had the closest relationship with *S. marcescens* strains SUStech OCE, By2Root2, TWV301, HBUR51222, and XC17 (Fig. 2). The isolate B.SB 1.1 was closely related to *S. marcescens* strain By2Root2, as evidenced by a 73% bootstrap value. The BLAST analysis of the deduced bacterial DNA sequences aligned isolate B.SB 1.1 most closely with the 16S rDNA sequence of *S. marcescens* strain SUStech OCE (Accession no. OP107019.1), showing high identity of 99.93% (Table 2).

### Phytochemical Test of Sea Fern Stem Extract and Endophytic Bacteria

Phytochemical test is a basic test to help determine the content of secondary metabolites produced by living organisms. Isolate B.SB 1.1 was selected for further testing because it contains pigments and is an endophytic bacterium of the genus *Serratia* from sea fern, and has not been reported previously. Phytochemical screening of the sea fern stem extract and crude extract of B.SB 1.1 isolate is presented in Table 3, and the screening results of the endophytic bacteria were in line with the secondary metabolites produced by the stem extract of the sea fern. This proves that endophytic bacteria isolated from plants are capable of producing the same secondary metabolites as their host plants. Endophytic bacteria are recognized for their capability to synthesize specialized metabolites or physiologically active compounds. Sea fern stem extract contains alkaloid, flavonoid, and saponin compounds, which are also found in isolate B.SB 1.1.

### α-Amylase Inhibitor Test from Endophytic Bacterial Metabolites

Endophytic bacterial isolate B.SB 1.1 was tested for α-amylase inhibitors as shown in Table 4. The α-amylase enzyme activity test was conducted to determine the ability of the α-amylase enzyme to hydrolyze starch into simple sugars. Strain B.SB 1.1 showed the percentage of inhibitor values at substrate concentrations of 1% and 2%. The inhibitor values obtained from B.SB 1.1 isolate showed positive results, demonstrating inhibition values of 0.34% and 32.57% against the α-amylase enzyme, approaching the control value (Acarbose). The results indicated that endophytic bacterial isolate B.SB 1.1 could become an antidiabetic drug candidate. This study has succeeded in discovering the potential of the symbiosis between endophytic bacteria and sea ferns in developing phytotherapeutic agents against diabetes.

### In Silico Analysis of Endophytic Bacteria Targeting α-Amylase

The preliminary screening in drug research and development, which includes estimating the biological activity of compounds using PASS Online, has given promising results. The LCMS extraction of sea fern stem endophytic isolate B.SB 1.1 has produced 88 compounds, 29 of which are predicted to have bioactivity and possess inhibitory activity against diabetes, with a greater probability of being active (Pa) than inactive (Pi) and Pa > 0.3. The Pa values of the 29 compounds as α-amylase inhibitors ranged from 0.307 to 0.629 (Table 5). The predicted bioactivity of these compounds, as determined by PASS Online, includes glucan endo-1,6-beta-glucosidase inhibitor, α-amylase inhibitor, beta-amylase inhibitor, antidiabetic symptomatic, antidiabetic, diabetic neuropathy treatment, diabetic retinopathy treatment, diabetic nephropathy treatment, and insulin promoter effects. Importantly, some of these compounds, such as BSB3 and BSB17, show potential as antidiabetic agents with five bioactivities related to antidiabetic effects, particularly as α-amylase inhibitors.

The bioactivity of these compounds, particularly the α-amylase inhibitors, are being tested for their pharmacokinetic properties and involves studying their absorption, distribution, metabolism, excretion, and toxicity (ADMET). The results of the ADMET study, which is a crucial aspect of computational drug design, are indicated in Supplementary 1, 2, and 3. Some compounds, such as BSB43, BSB47, BSB53, BSB56, and BSB72, meet all absorption parameters. The water solubility of endophytic bacteria compounds ranges from -7.728 to 0.033. High CaCo_2_ permeability with a value > 0.90 predicts the compounds have high intestinal mucosa permeability. Based on pkCSM, some compounds, such as BSB3, BSB24, BSB39, BSB42, BSB60, BSB62, BSB63, BSB79, and BSB84, have low CaCo2 permeability, while the others have high Caco2 permeability. Additionally, all compounds have positive human intestine absorption (HIA) of more than 30%, suggesting they can be easily absorbed in the human intestine. These findings also indicate promising potential compounds as drugs, as they are not substrates or inhibitors of P-glycoprotein.

Twelve compounds, identified as BSB8, BSB10, BSB13, BSB18, BSB21, BSB31, BSB47, BSB48, BSB53, BSB56, BSB59, and BSB72, satisfied all criteria pertaining to distribution parameters. Only unbound (free) drug molecules are capable of engaging with pharmacological target proteins, endophytic bacterial compounds demonstrating unbound fraction values ranging from 0 to 0.833. Furthermore, the permeability of the blood-brain barrier (BBB) and central nervous system (CNS) assumes critical significance. Chemical compounds exhibiting a logBB value exceeding 0.3 are posited to effectively traverse the BBB, whereas those with a logPS value greater than 3 are deemed capable of penetrating the CNS.

The metabolism parameters are substrates or inhibitors of the cytochrome P450 (CYP450) enzyme family. Compounds BSB3, BSB21, BSB24, BSB40, BSB44, BSB63, BSB67, BSB72, and BSB79 do not act as substrates or inhibitors of CYP450, so these compounds will not interfere with the metabolic process of drugs in the body. Total clearance, which combines hepatic and renal clearance, is a crucial factor in determining the dose level required to achieve stable concentrations. The highest total clearance indicates that the drug is excreted from the body faster. Compound BSB84 has the highest total clearance at 2.518. Only one compound, BSB59, is predicted to act as a renal OCT2 substrate.

A crucial factor in pharmacokinetics is toxicity. Compounds that exhibit toxicity cannot be developed as drugs due to their harmful nature. Parameters such as mutagenicity (AMES and Ptotox-II), maximum rate tolerance dose (MRTD), oral rat acute toxicity (ORAT), hepatotoxicity, carcinogenicity, and lethal dose (LD50) are essential in evaluating toxicity. The maximum rate tolerance dose of the tested compounds ranged from -0.755 to 1.456 mg/kg/day. Out of the 29 compounds tested, 17 were found to be non-toxic based on pkCSM and Protox-II, indicating potential for drug development. However, three of them were categorized as highly toxic (BSB42) and moderately toxic (BSB17, BSB44) based on the LD50 classification (mg/kg) with LD50 values ranging from 48-250 mg/kg.

Fourteen endophytic bacterial compounds from sea fern stems were filtered based on ADMET parameters and toxicity testing with AMES and Protox-II. The compounds that pass these initial tests will be analyzed for drug-likeness based on Lipinski's rule. Lipinski's rule includes criteria such as molecular weight less than or equal to 500 Da, no more than 5 H-bond donors, no more than 10 H-bond acceptors, and a log P value less than or equal to 5. Based on the drug-likeness analysis results, we found that only one compound (BSB39) did not meet Lipinski's rule criteria. In contrast, the others met the criteria and had a high bioavailability score of 0.55 (Table 6). Compounds that satisfied all five drug-likeness filters significantly differed from compounds that did not fulfill Lipinski's rule.

**Fig. 3:**
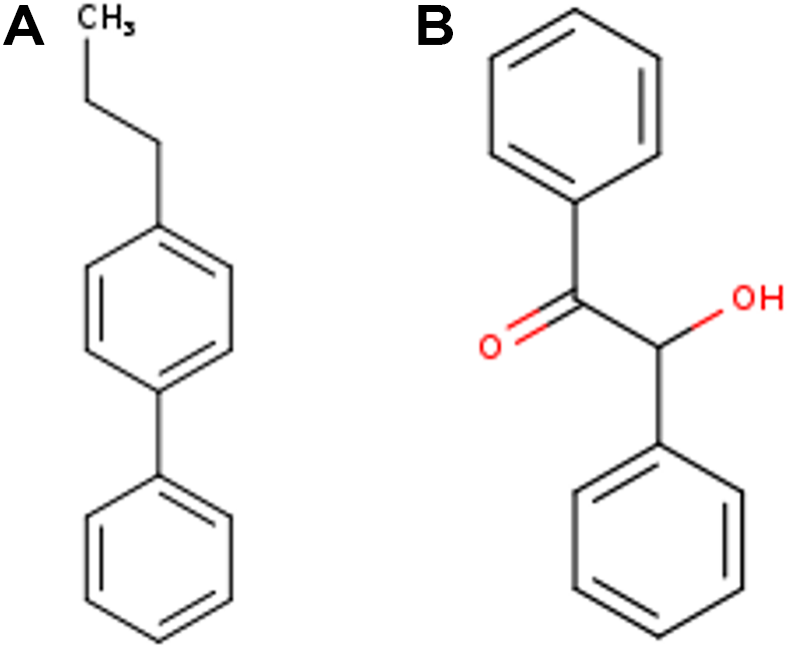
Chemical structures of the compounds B.SB 1.1. (**A**) 4-Propylbiphenyl and (**B**) Benzoin.

**Fig. 4:**
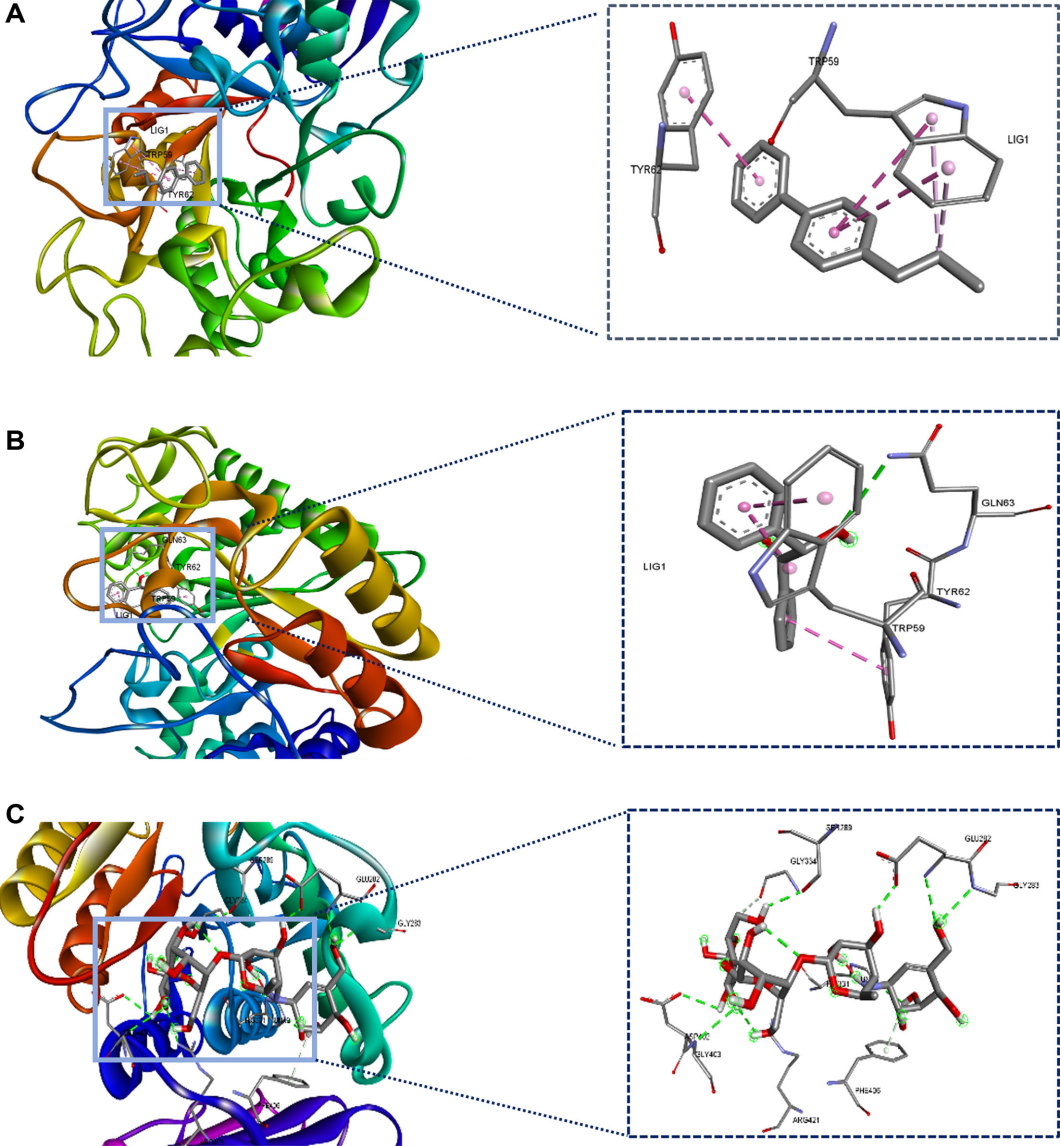
Interactions the compounds with α-amylase enzyme. (**A**) The interaction of 4-Propylbiphenyl-BSB10 with α-amylase shows hydrophobic interaction; (**B**) The interaction of Benzoin-BSB8 with with α-amylase show hydrogen-bonded interaction and hydrophobic interaction; (**C**) The interaction of Acarbose (Native ligand) with α-amylase show only hydrogenbonded interaction.

Thirteen compounds that met the pharmacokinetics and Lipinski rule criteria were selected for molecular docking. These compounds have a range of binding energy values from -2.9 to -7.6 (see Table 7). The compounds exhibited good binding energy, with the lowest being for 4-Propylbiphenyl-BSB10 (-7.6 kcal/mol) and Benzoin-BSB8 (-7.5 kcal/mol). These binding energy values are close to the value of the standard inhibitor, Acarbose (-8.2 kcal/mol). The structures of the compounds 4-propylbiphenyl and benzoin are presented in Fig. 3. A comparison of the binding poses (3D) and interactions of two compounds, BSB8 and BSB10, with the α-amylase enzyme and the native ligand (Acarbose) is presented in Fig. 4. According to Table 7 and Fig. 4, BSB8 forms one hydrogen-bonded interaction with GLN63 and hydrophobic interactions with TYR62 and TRP59. On the other hand, BSB10 does not exhibit hydrogen bonding and only shows hydrophobic interactions with TYR62 and TRP59. Furthermore, among the screened compounds, BSB8 and BSB10 have RMSD values lower than 1.00 Å, which are smaller than the RMSD value of Acarbose. Specifically, BSB8 has an RMSD value of 0.444, and BSB10 has a value of 0.141. This study represents the first report on the antidiabetic potential of the metabolites 4-propylbiphenyl and benzoin, derived from endophytic bacteria of sea fern stems, through their interaction with the α-amylase enzyme. This represents an important strategy for controlling postprandial hyperglycemia.

## Discussion

Isolation and identification of endophytic bacteria was performed from the stems of sea ferns (*Acrostichum aureum* L.) grown on the east coast of Sumatra Island, Indonesia, precisely in Selatbaru Village, Bengkalis Regency, Riau Province. Endophytic bacteria of sea fern stems were identified by morphology technique. In our study, the endophytic bacteria from this sea fern stems were gram-negative. Several studies have shown that the quantity of gram-negative bacteria equals those that are gram-positive. Nevertheless, gram-negative endophytic bacteria were more abundant than gram-positive ones [37].

Based on identification by 16S ribosomal RNA (rRNA) gene sequences, isolate B.SB 1.1 showed the highest sequence homology to *S. marcescens*, a species whose members are rod-shaped, gram-negative bacteria belonging to the Enterobacteriaceae family, which is recognized for synthesizing prodigiosin, and a distinctive red pigment [38]. The characteristics of isolate B.SB 1.1, as delineated through our research, are consistent with those of *S. marcescens*, notably its production of a purplish-red, gram-negative pigment and its bacil (rod-shaped) morphology, which substantiates its classification within the *S. marcescens* species. *S. marcescens* is ubiquitously distributed, having been isolated from diverse environments, including water, soil, plants, and insects [39-40]. Remarkably, *S. marcescens* has been identified in an array of plant species, such as rice [41], *Vaccinium uliginosum* leaves [42], tomatoes [43], cactus [44], and *Bryophyllum pinnatum* [45]. To the best of our knowledge, this is the first study to report the isolation of endophytic bacteria from *Acrostichum aureum* L. similar to *Serratia* species.

*Serratia* species are capable of synthesizing a variety of chemical compounds with antibacterial capabilities [46]. Furthermore, several strains of *S. marcescens* have been identified as plant endophytes, which can inhibit phytopathogens and enhance crop growth [47-49]. Additionally, *S. marcescens* is known to produce chitinases, glucanases, cellulases, and the pigment prodigiosin, which could serve as antifungal agents [50].

Here, we found isolates from stems of sea fern that contain alkaloids, flavonoids and saponin, which are also found in sea fern stems. These endophytes are instrumental in the biosynthesis of bioactive compounds that facilitate the host plant's systemic resistance to infectious agents. This dynamic symbiosis underscores the potential of endophytic bacteria in the sustainable production of therapeutically valuable compounds. Such compounds have found significant applications in the pharmaceutical industry, serving as the basis for the development of antibiotics, anti-cancer agents, antiviral, anti-diabetic, and other bioactive molecules [51]. Furthermore, endophytic bacteria, through long-term association with plant tissues, tend to establish symbiotic relationships, wherein these bacteria adapt their gene expression to synthesize the secondary metabolites required by their host plants [15].

The stems of the sea fern (*Acrostichum aureum* L.) extracted with methanol and ethanol solvents from the Tamil Nadu region, India, produced saponins and steroid compounds [52]. Alkaloids are also found in endophytic bacteria from the sterile leafs of sea fern (*Acrostichum aureum*) [18]. The nutrient content of the soil in each sampling area affects the phytochemical content of plants and their endophytic bacteria. Differences in the results of the phytochemical tests are also thought to be caused by differences in solvents and methods that affect the crude extracts of the secondary metabolites produced [17].

Endophytic bacteria from *Pinellia ternata* such as *B. cereus*, *Aranicola proteolyticus*, *Serratia liquefaciens*, *Bacillus thuringiensis*, and *Bacillus licheniformis* also produce alkaloids identical to their host plant [53]. Alkaloids are essential in human medicine as anesthetics, cardioprotective, anti-inflammatory agents, and anti-diabetes drugs [54-55]. Flavonoids act as antioxidants [56]. *Staphylococcus caprae* is an endophytic bacterium from *Mimosa pudica* leaf that is reported to have flavonoids against SARS-COV-2 [57]. Saponin is also produced by the endophytic bacteria *S. marcescens* strain MBC1 [58].

Endophytic bacteria from medical plants are an essential source of secondary metabolites and enzymes valuable to the pharmaceutical industry [16]. Therefore, many researchers are conducting studies to characterize and identify such bacteria derived from a wide range of medicinal plants. Some endophytic bacteria are known to have antidiabetic activity, such as the endophytic bacteria from rubber plants (*Hevea brasiliensis*) [59], and those from stems and roots of *A. aureum* Meranti islands [60].

In the current investigation, we tested the ability of isolate B.SB 1.1 as an α-amylase inhibitors α-amylase inhibitors are compounds that can slow glucose absorption by inhibiting the action of carbohydrate hydrolyzing enzymes such as α-amylase. These compounds can control blood sugar levels in people with diabetes mellitus (DM) [61]. The results show endophytic bacteria from stems of sea fern could inhibit α-amylase enzyme activity with a value of 32.57%, which approaches the control Acarbose value. The endophytic bacteria had different inhibitions to α-amylase. Endophytic bacteria from the roots of *Rhizophora stylosa* show α-amylase inhibitory with values ranging from 31.4 to 59.7% [62]. Other endophytic bacteria from sterile leaf of *A. aureum* have an inhibitor value of 18.12 % [18], and *Hevea Brasiliensis* has an inhibitor value of 29.44% [59]. One way to treat diabetes mellitus is by inhibiting the work of enzymes that hydrolyze carbohydrates to reduce glucose absorption. The α-amylase enzyme plays a vital role in breaking oligosaccharides and disaccharides into monosaccharides for absorption [63]. Inhibition of the α-amylase enzyme can delay and prolong the digestion of carbohydrates, causing a decrease in the rate of glucose absorption and preventing an increase in postprandial plasma glucose levels [64].

In silico studies are needed for drug development and pharmacokinetics testing is needed to know how the body interacts with administered substances for the entire duration of exposure, including absorption, distribution, metabolism, excretion, and toxicity. Water solubility is a key that significantly influences absorption [65-66]. Another parameter for absorption is substrate or inhibitor P-glycoprotein. Compounds that act as substrates or inhibitors of P-glycoprotein will interfere with the drug absorption process. P-glycoprotein plays a crucial role in protecting cells from toxic compounds and can reduce the absorption activity of a drug compound [67]. Distribution is a parameter instrumental in delineating the pharmacokinetics of drugs within the human body, thus influencing their efficacy, safety, and the optimization of dosing schedules through mechanisms such as diffusion and convection [68]. The steady-state volume of distribution (VDss) emerges as a pivotal metric, elucidating that a compound exhibits a greater proclivity for distribution within bodily tissues as compared to its plasma concentration [69].

The metabolism parameters are substrates or inhibitors of the cytochrome P450 (CYP450) enzyme family. The CYP450 isoforms, particularly CYP1A2, CYP2C9, CYP2C19, CYP2D6, and CYP3A4, are responsible for a significant 90% of drug metabolism [70]. These CYP450 isoforms play a crucial role in inactivating and activating drugs [71]. Excretion is the process of eliminating drugs from body whose parameters are total clearance and renal OCT2 substrate. Organic cation transporter 2 (OCT2) is a transporter in the human kidney that controls the reuptake of drugs from the blood, playing a vital role in the renal disposition and clearance of drugs [72]. Understanding the impact of OCT2 on drug clearance is crucial for predicting drug interactions and ensuring safe and effective drug use. Some compounds are classified as toxic or hazardous. Compounds with LD50 values less than or equal to 300 mg/kg are classified as hazardous [73], which means BSB42, BSB17, and BSB44 compounds are not recommended for drug development. This highlights the significance of non-toxic compounds in the development of new drugs.

The next step in drug development is in silico analysis for drug-likeness using Lipinski’s rules. Compounds that do not meet Lipinski’s criteria are predicted to have poor absorption or permeation [74]. The bioavailability score also helps to identify poorly and well-absorbed compounds [65].

Based on the molecular docking, 4-propylbiphenyl and benzoin have the lowest energy binding to α-amylase enzyme with values of -7.6 and -7.5 kcl/mol. The lower the binding affinity energy value of a compound to the target protein, the better the compound as a drug candidate. Compounds of endophyte extract from *Leucas ciliata* have binding affinity with α-amylase ranging from -4.7 to -10.1 kcl/mol [75], while novel enantiopure isoxazolidine compounds have binding affinity ranging from -3.3 to -5.6 kcl/mol [76].

Several benzoin derivatives from fungal endophytes are reported to exhibit antidiabetic activity [77]. Benzoin compounds are also reported to possess activity against α-amylase and a-glucosidase, indicating that they have potential as antidiabetic agents [78]. In addition, 4-propylbiphenyl has not been reported to have significant pharmacological potency or therapeutic applications, and there is limited information on its pharmacological potency. Based on bioactivity predictions using PASS Online, 4-propylbiphenyl acts as a glucan endo-1,6-beta-glucosidase inhibitor, α-amylase inhibitor, diabetic neuropathy treatment, and insulin promoter. These bioactivities are closely related to antidiabetic properties. Likewise, benzoin is a candidate α-amylase inhibitor that has not been reported in previous research. Benzoin compounds from endophytic bacteria of sea fern stems have bioactivity that acts as a glucan endo-1,6-beta-glucosidase inhibitor, α-amylase inhibitor, beta-amylase inhibitor, and diabetic neuropathy treatment based on PASS Online. Benzoin has been reported to act as an anticancer agent, inhibiting cell proliferation and inducing apoptosis in human colon carcinoma cells [79]. Furthermore, 4-propylbiphenyl and benzoin are natural inhibitor candidates from endophytic bacteria of sea ferns, a fact supported by their low binding energy value. These compounds also have hydrogen and hydrophobic interaction. Hydrogen bonding and hydrophobic interactions are crucial for the stability of compound bonding and for enhancing potency towards the target, thereby contributing to drug efficacy [80]. The RMSD value of 4-propylbiphenyl and benzoin is lower than that of Acarbose, thus showing these compounds are more stable when binding with α-amylase. The RMSD value represents the conformational response of the complex over time, with consistently low values indicating that the ligand structure remains similar to the docking posture, and fluctuating values indicate regular changes [27].

## Conclusion

Endophytic bacteria *S. marcescens* B.SB 1.1 showed presence of the same secondary metabolites in the crude extracts and host plant, demonstrating significant pharmacological potential. In vitro assays revealed that *S. marcescens* B.SB 1.1 exhibited α-amylase inhibition at 32.57% with a 2% starch substrate concentration, approaching the inhibitory effect of the standard antidiabetic drug Acarbose (47.09%). In silico docking studies identified two compounds, 4-propylbiphenyl and benzoin, as having the lowest binding energies to the α-amylase enzyme. These findings indicate that *S. marcescens* B.SB 1.1 has potential as an antidiabetic agent.

## Supplemental Materials

Supplementary data for this paper are available on-line only at http://jmb.or.kr.



## Figures and Tables

**Table 1:** The characteristic of endophytic bacterial isolates from sea fern stems (*A. aureum* L.).

Isolate Code	Morphology of colony				Morphology cell	
	Colour	Edge	Elevation	Size	Shape	Gram
B.SB 1.1	Purplish red	Entire	Convex	Small	Bacil	-

**Table 2:** NCBI BLAST 16S rRNA gene sequences closest related species with endophytic bacterial isolated from sea fern stems (*A. aureum* L.).

Species Name	Max Score	Query Cover	E value	Ident	Accession
*Serratia marcescens* strain SUStech OCE	2595	100%	0.0	99.93%	OP107019.1
*Serratia marcescens* strain By2Root2	2590	100%	0.0	99.86%	KM099141.1
*Serratia marcescens* strainTWV301	2590	100%	0.0	99.86%	MW713800.1
*Serratia marcescens* strain HBUR51222	2590	100%	0.0	99.86%	OR502274.1
*Serratia marcescens* strain XC17	2588	100%	0.0	99.86%	PP471167.1

**Table 3:** Phytochemical screening of sea fern stems (*Acrostichum aureum* L.) extract and endophytic bacteria crude extracts.

Samples	Phytochemical test					
	Alkaloids	Flavonoids	Saponins	Tannins	Steroids	Terpenoids
Sea fern stems extract	+	+	+	-	-	-
B.SB 1.1	+	+	+	-	-	-

Note: + = Identified; - = not identified

**Table 4:** Inhibitors of α-amylase of endophytic bacteria.

Isolates	Starch concentration	Inhibition Value (%)
B.SB 1.1	1 %	0.34
	2 %	32.57
Acarbose (A)	1%	21.43
	2%	47.09

**Table 5:** Biological activity prediction of endophytic bacteria using PASS Online.

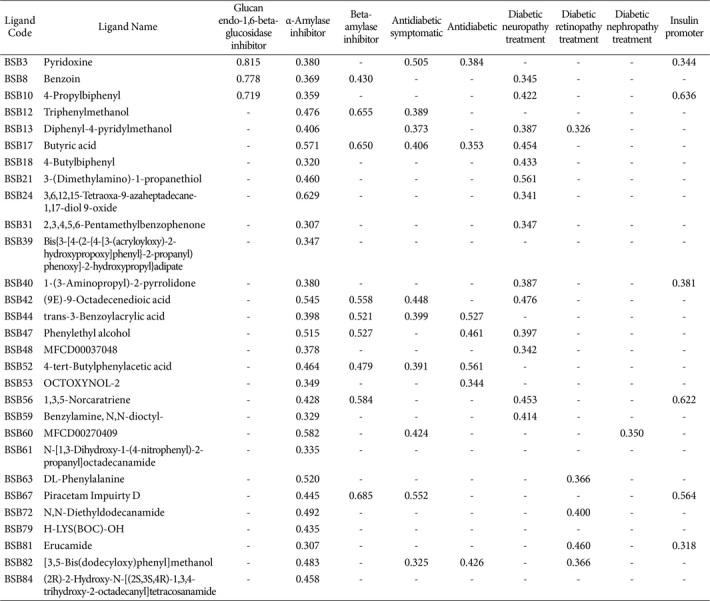

**Table 6:** Drug-likeness prediction and bioavailability of endophytic bacteria.

Ligands Code	Lipinski’s rule				Bioavailability	
	MW	HBA	HBD	MLOGP	Value	Status
BSB3	169.18	4	3	-0.91	0.55	High
BSB8	212.24	2	1	2.33	0.55	High
BSB10	196.29	0	0	5.58	0.55	High
BSB21	119.23	1	0	1.16	0.55	High
BSB39*	971.09	16	4	2.45	0.17	Low
BSB40	142.20	2	1	-0.05	0.55	High
BSB47	122.16	1	1	1.87	0.55	High
BSB48	267.45	2	0	4.33	0.55	High
BSB53	294.43	3	1	3.05	0.55	High
BSB60	354.52	4	2	4.70	0.55	High
BSB72	255.44	1	0	3.79	0.55	High
BSB79	246.30	5	3	-1.61	0.55	High
BSB81	337.58	1	1	5.06	0.55	High
BSB82	476.77	3	1	6.03	0.55	High

*Compounds doesn’t meet parameters. MW = molecular weight (≤500 g/mol), HBA (N or O) = hydrogen bond acceptor (≤10), HBD (NH or OH) = hydrogen bond donor (≤5), MLOGP = lipopolycity.

**Table 7:** Binding affinity for the selected compounds of endophytic bacteria.