# Supplementary material for: Efficacy of Endophytic Bacterium Serratia marcescens B.SB 1.1 associated with Sea Fern (Acrostichum aureum L.) as an Antidiabetic Agent
Source: J Microbiol Biotechnol. 2025 Apr 27;35:e2412031. doi: 10.4014/jmb.2412.12031 (PMC12089956; doi:10.4014/jmb.2412.12031)
Supplement: Supplementary file 1 [file jmb-35-e2412031-supple.pdf]

J. Microbiol. Biotechnol. 2025. 35: e2412031

https://doi.org/10.4014/jmb.2412.12031

Supplementary Tables

Table S1. Absorbtion and distribution parameters of endophytic bacteria compounds.

Ligand Codes	Water solubility	Caco-2 Permeability	Intestinal Absorption	P-glycoprotein Substrate	P-glycoprotein I Inhibitor	P-glycoprotein II Inhibitor	VDss	Fraction Unbound	BBB Permeability	CNS Permeability
**Expected value**	**log mol/L**	**>0.9**	**>30%**	**No**	**No**	**No**	**> -0.15**	**Fu**	**> -0.3**	**> -3**
BSB 3	-2.108	0.561	94.139	No	No	No	0.589	0.819	-0.893	-3.364
BSB 8	-3.098	1.357	95.116	Yes	No	No	0.339	0.166	0.383	-1.694
BSB 10	-5.278	1.59	95.802	Yes	No	No	0.823	0.026	0.642	-1.267
BSB 12	-6.194	1.593	97.681	Yes	No	No	-0.401	0.05	1.152	-1.43
BSB 13	-4.845	1.748	97.684	Yes	No	No	0.16	0.111	0.712	-1.599
BSB 17	-0.19	1.562	92.202	No	No	No	-0.831	0.685	-0.258	-2.537
BSB 18	-5.595	1.59	95.471	Yes	No	No	0.922	0.01	0.689	-1.269
BSB 21	-0.567	1.363	97.206	Yes	No	No	0.397	0.747	0.203	-2.578
BSB 24	-1.376	-0.438	41.864	No	No	No	-0.692	0.881	-1.105	-3.859
BSB 31	-5.127	1.189	97.01	Yes	No	No	0.633	0.044	0.553	-1.329
BSB 39	-2.976	-0.216	58.417	Yes	Yes	Yes	-1.67	0.422	0.422	-4.198
BSB 40	0.033	1.136	86.546	No	No	No	0.399	0.833	-0.22	-3.05
BSB 42	-3.298	0.252	93.188	No	No	No	-1.452	0.121	-0.441	-3.007
BSB 44	-1.989	1.217	95.32	No	No	No	-0.996	0.417	-0.167	-2.382
BSB 47	-1.198	1.605	88.073	No	No	No	0.202	0.434	-0.089	-1.857
BSB 48	-7.219	1.465	91.962	No	No	No	0.44	0.072	0.713	-1.725
BSB 52	-3.179	1.679	96.868	No	No	No	-1.131	0.227	0.382	-1.523
BSB 53	-4.299	1.852	93.461	No	No	No	0.373	0.113	-0.016	-2.075
BSB 56	-2.17	1.522	95.591	No	No	No	0.427	0.378	0.447	-1.555
BSB 59	-5.957	1.148	87.267	Yes	Yes	No	1.662	0.038	1.316	-0.68
BSB 60	-5.888	0.417	91.247	No	Yes	Yes	-0.334	0.124	-0.857	-3.26

BSB 61	-6.097	0.675	77.081	Yes	Yes	Yes	0.046	0.038	-1.066	-3.179
BSB 63	-2.89	0.62	76.21	No	No	No	-0.326	0.492	-0.271	-2.675
BSB 67	-0.306	1.093	87.035	No	No	No	-0.732	0.762	-0.294	-3.075
BSB 72	-5.974	1.476	90.736	No	No	No	0.438	0.19	0.757	-2.291
BSB 79	-2.888	-0.264	43.894	Yes	No	No	-0.552	0.445	-0.845	-3.448
BSB 81	-7.728	1.104	88.843	No	No	Yes	0.173	0.021	-0.558	-1.432
BSB 82	-6.093	1.167	86.143	No	Yes	Yes	0.491	0	-0.674	-1.442
BSB 84	-2.9	0.398	63.666	Yes	Yes	Yes	-0.986	0.296	-2.47	-3.736

Table S2. Metabolism and Excretion parameters of endophytic bacteria compounds.

Ligand Codes	CYP2D6 Substrate	CYP3A4 substrate	CYP1A2 inhibitor	CYP2C19 inhibitor	CYP2C9 inhibitor	CYP2D6 inhibitor	CYP3A4 inhibitor	Total Clearance	Renal OCT2 Substrate
**Expected value**	**No**	**No**	**No**	**No**	**No**	**No**	**No**	**Log(ml/min/kg)**	**No**
BSB 3	No	No	No	No	No	No	No	0.688	No
BSB 8	No	No	Yes	Yes	No	No	No	0.191	No
BSB 10	No	Yes	Yes	Yes	Yes	No	No	0.253	No
BSB 12	No	Yes	Yes	Yes	Yes	No	No	0.1	No
BSB 13	No	Yes	Yes	Yes	Yes	No	Yes	0.174	No
BSB 17	No	No	No	No	No	No	No	0.416	No
BSB 18	No	Yes	Yes	Yes	Yes	No	No	0.267	No
BSB 21	No	No	No	No	No	No	No	1.049	No
BSB 24	No	No	No	No	No	No	No	1.338	No
BSB 31	No	Yes	Yes	Yes	Yes	No	No	0.337	No
BSB 39	No	Yes	No	No	No	No	Yes	0.607	No
BSB 40	No	No	No	No	No	No	No	1.088	No
BSB 42	No	Yes	No	No	No	No	No	1.835	No
BSB 44	No	No	No	No	No	No	No	0.706	No
BSB 47	No	No	Yes	No	No	No	No	0.325	No
BSB 48	No	Yes	Yes	Yes	No	No	No	2.069	No
BSB 52	No	No	No	No	No	No	No	0.204	No
BSB 53	No	Yes	Yes	Yes	No	No	No	1.054	No
BSB 56	No	No	Yes	No	No	No	No	0.164	No
BSB 59	Yes	Yes	No	No	No	Yes	No	1.525	Yes

BSB 60	No	Yes	Yes	No	No	No	No	2.136	No
BSB 61	No	Yes	No	Yes	No	No	Yes	1.692	No
BSB 63	No	No	No	No	No	No	No	0.452	No
BSB 67	No	No	No	No	No	No	No	0.601	No
BSB 72	No	No	No	No	No	No	No	1.925	No
BSB 79	No	No	No	No	No	No	No	0.232	No
BSB 81	No	Yes	Yes	No	No	No	No	2.09	No
BSB 82	No	Yes	No	No	No	No	No	1.84	No
BSB 84	Yes	Yes	No	No	No	No	No	2.518	No

Table S3. Toxicity parameters of endophytic bacteria compounds.

Compound	AMES Toxicity	MRTD	ORAT	Hepatotoxicity	Carcinogenetic	Mutagenecity	LD50	LD50 class
**Expected value**	**No**	**mg/kg/day**	**mol/kg**	**No**	**Inactive**	**Inactive**	**mg/kg**	
BSB 3	No	-0.18	1.852	No	Inactive	Inactive	3350	5
BSB 8	No	0.694	1.983	No	Inactive	Inactive	3000	5
BSB 10	No	0.841	1.887	No	Inactive	Inactive	2400	5
BSB 12	No	0.48	1.69	Yes	Inactive	Inactive	5000	5
BSB 13	Yes	0.136	1.794	No	Inactive	Inactive	250	3
BSB 17	No	0.91	1.72	No	Inactive	Inactive	91	3
BSB 18	No	0.884	1.897	Yes	Inactive	Inactive	2400	5
BSB 21	No	0.921	2.29	No	Inactive	Inactive	1050	4
BSB 24	No	1.144	1.421	No	Active	Active	560	4
BSB 31	Yes	0.636	2.099	Yes	Inactive	Inactive	2795	5
BSB 39	No	0.386	2.621	No	Inactive	Inactive	3000	5
BSB 40	No	0.761	2.303	No	Inactive	Inactive	2900	5
BSB 42	No	-0.946	2.02	No	Inactive	Inactive	48	2
BSB 44	No	1.456	1.94	No	Inactive	Inactive	250	3
BSB 47	No	0.98	1.881	No	Inactive	Inactive	800	4
BSB 48	No	0.018	1.765	No	Inactive	Inactive	1600	4
BSB 52	No	0.638	2.218	Yes	Inactive	Inactive	670	4
BSB 53	No	0.687	2.12	No	Inactive	Inactive	2800	5
BSB 56	No	0.751	1.824	No	Active	Inactive	2250	5
BSB 59	No	0.27	2.332	Yes	Inactive	Inactive	230	3
BSB 60	No	0.09	1.717	No	Inactive	Inactive	39800	6

BSB 61	No	-0.755	3.828	Yes	Inactive	Active	2640	5
BSB 63	No	0.935	2.193	Yes	Inactive	Inactive	2400	5
BSB 67	No	1.326	1.612	No	Active	Inactive	6090	6
BSB 72	No	0.115	1.919	No	Inactive	Inactive	3200	5
BSB 79	No	1.116	2.122	No	Inactive	Inactive	1000	4
BSB 81	No	-0.469	1.873	No	Inactive	Inactive	750	4
BSB 82	No	0.309	1.358	N0	Inactive	Inactive	1414	4
BSB 84	No	0.188	2.536	Yes	Inactive	Inactive	5000	5

* MRTD: Maximum rate tolerance dosis, ORAT: Oral rat acute toxicity
